# Influence of the Amount of Toothpaste on Cleaning Efficacy: An *In Vitro* Study

**DOI:** 10.1055/s-0042-1747953

**Published:** 2022-07-04

**Authors:** Sandra Sarembe, Carolin Ufer, Andreas Kiesow, Hardy Limeback, Frederic Meyer, Ines Fuhrmann, Joachim Enax

**Affiliations:** 1 Fraunhofer Institute for Microstructure of Materials and Systems IMWS, Halle, Germany; 2 Faculty of Dentistry, University of Toronto, Toronto, Ontario, Canada; 3 Research Department, Dr. Kurt Wolff GmbH & Co. KG, Bielefeld, Germany

**Keywords:** brushing machine, cleaning efficacy, enamel, teeth, toothpaste

## Abstract

**Objectives** The aim of this *in vitro* study was to test the influence of the amount of toothpaste on enamel cleaning efficacy.

**Materials and Methods** The hydrated silica-based test toothpaste (radioactive dentin abrasion: 60.19 ± 1.35) contained all ingredients of a regular fluoride toothpaste. The cleaning efficacy of four different toothpaste amounts (1.00 g, 0.50 g [both “full length of brush”], 0.25 g [“pea-size”], and 0.125 g [“grain of rice-size”]) diluted in 1.00 mL water were each tested for different brushing times (10, 30, 60, 120, 180, and 300 seconds) using a standardized staining model on human molars with a brushing machine. Photographic documentation and colorimetric measurements were conducted, respectively, initially, after staining and after each brushing step. Colorimetric measurements were used to calculate the stain removal (in %).

**Statistical Analysis** Results were analyzed by one-way analysis of variance with post hoc Tukey test and Levene's test for analysis of homogeneity of variance. The level of significance *α* was set at ≤ 0.05.

**Results** The cleaning efficacy decreased significantly when using smaller toothpaste amounts. Stain removal after 120 seconds brushing time was: 77.4 ± 5.0% (1.00 g toothpaste), 75.7 ± 3.4% (0.50 g toothpaste), 54.1 ± 6.7% (0.25 g toothpaste), and 48.2 ± 7.1% (0.125 g toothpaste), respectively.

**Conclusion** In this *in vitro* study the cleaning efficacy of a medium-abrasive, hydrated silica-based toothpaste was analyzed. Note that 1.00 g toothpaste showed for all brushing times a significantly higher cleaning efficacy than 0.25 g toothpaste and 0.125 g toothpaste.

## Introduction

Caries and gum disease (gingivitis/periodontitis/peri-implantitis) are plaque-associated diseases. 1 2 3 Thus, one cornerstone in daily oral care is to remove dental plaque as effectively as possible. 4 For that, the market offers various toothpaste formulations. 5 6 Most important for cleaning effectiveness of any toothpaste is the addition of particulate abrasives. 5 7 8 9 Many different abrasives are used in toothpastes, for example, hydrated silica, calcium carbonate, calcium phosphates, perlite, alumina, and sodium bicarbonate. 9 10 11 12 Hydrated silica and calcium carbonate are used in concentrations of up to 20%. 5 Different abrasives differ in relative hardness values and consequently in their cleaning efficacy and abrasion properties. 9 Perlite and alumina, for example, are used as polishing agents due to their hardness and are used in limited concentrations of approximately 1 to 2%. 5 9 In some toothpaste formulations for tooth whitening different abrasives are combined. 13 In general, there is a trend of improvement to highly efficient abrasives, which show good plaque disruption or removal abilities, while having reduced radioactive dentin abrasion (RDA) values. 14 15

In addition to delivering therapeutic agents to combat caries and gingivitis, toothpastes are formulated to remove dental plaque, as well as stains, as effectively as possible while being gentle to teeth and gingiva. 5 16 17

Toothpastes are highly complex semisolid pastes that contain several active ingredients. One well-known active ingredient for remineralization and caries prevention is fluoride. 18 19 Due to regulatory reasons on the use of fluoride toothpastes for children the amount of fluoride toothpaste, which can be applied, is limited. For example, for children up to 2 years the size of a grain of rice (0.125 g) and for children of 2 to 6 years a pea size (0.25 g) of fluoride toothpaste is recommended by the European Academy of Paediatric Dentistry. 20 The regulation of the European Parliament and of the Council on cosmetic products prescribes that children of 6 years and younger should use a pea-sized amount of toothpaste if the toothpaste contains 0.1 to 0.15% fluoride. 21

Additionally, some toothbrush heads, particularly electric brushes, have become smaller, so also adults may be using lower amounts of toothpaste. 22 As well, the use of tap water to wet the toothbrush and/or the toothpaste may influence toothpaste dilution. 23

To date, to the best of the authors' knowledge, no study has analyzed the influence of the toothpaste amount on cleaning efficacy. Therefore, the aim of this *in vitro* study was to analyze the influence of different toothpaste amounts (e.g. “grain of rice,” “pea,” “full length of brush” 20 ) and dilutions on the cleaning efficacy, respectively. These new data will help to understand to which extend stain and plaque removal efficacy can be expected using different toothpaste amounts *in vivo* .

## Materials and Methods

### Sample Preparation

Thirty-two enamel specimens were derived from extracted sound human molars. The enamel specimens were embedded in epoxy resin (EpoFix, Struers, United States) and ground to 1,200 grit (Struers). To facilitate the adhesion of the polyphenols during storage in the staining media, an etching procedure was included. The samples were slightly etched by immersing in 1% hydrochloric acid solution (1 minute, Carl Roth, Germany), followed by saturated sodium carbonate solution (2 minutes, Acros Organics B.V.B.A, Germany) and 1% phytic acid solution (1 minute; Acros Organics B.V.B.A, Germany). 14

### Test Toothpaste

The test toothpaste (an experimental toothpaste; not commercially available) had a RDA value of 60.19 ± 1.35 (data on file; RDA measurement according to International Organization for Standardization [ISO] 11609 and American National Standards Institute/American Dental Association [ADA] Standard No. 130; performed by Therametric Technologies, Inc., United States). Note that ISO/ADA reference material (calcium pyrophosphate powder; RDA standard grade, Odontex Inc., Lawrence, Kansas, United States) was used for testing.

The toothpaste composition was as follows:

Aqua, Hydrated Silica, Glycerin, Hydrogenated Starch Hydrolysate, Xylitol, Silica, Cellulose Gum, Sodium Sulfate, Sodium Methyl Cocoyl Taurate, 1,2-Hexanediol, Aroma, Caprylyl Glycol, Sodium Fluoride (1450 ppm F ^-^ ), Sodium Cocoyl Glycinate.

The stain removal properties of four different toothpaste amounts each diluted in 1 mL water (to simulate a dilution by saliva during tooth brushing) were investigated on eight tooth samples in each group ( *n* = 8) ( Table 1 ).

**Table 1:** Overview of toothpaste amounts used for this study

Toothpaste amount		Dilution
1.00 g	Corresponding to a “full length of brush” amount; max. (over 6 years)	1.00 g toothpaste : 1.00 mL water
0.50 g	Corresponding to a “full length of brush” amount; min. (over 6 years)	0.50 g toothpaste : 1.00 mL water
0.25 g	Corresponding to a “pea-size” amount (2–6 years)	0.25 g toothpaste : 1.00 mL water
0.125 g	Corresponding to a “grain of rice-size” amount” (First tooth, up to 2 years)	0.125 g toothpaste : 1.00 mL water

Note: **T** he toothpaste amounts were taken from Toumba et al. 20

### Teeth Staining

**Fig. 1:**
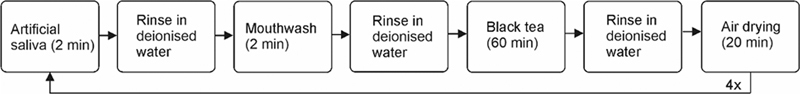
Overview about the staining procedure used in this study.

The cleaning efficacy was tested by means of stain removal. Tooth samples were stained according to a modified protocol from Lath et al 24 (a highly standardized and reproducible staining model). The staining procedure is depicted in Fig. 1 . Specimens were rinsed with artificial saliva (2 minutes at room temperature) and with deionized water, exposed to chlorhexidine containing mouthwash (2 minutes, Chlorhexamed Forte 0.2%, GSK, at room temperature) and rinsed with deionized water again. The samples were then placed in warm black tea (60 minutes at 37°C) and a subsequent rinsing step with deionized water. Finally, the samples were air-dried (20 minutes). In total, four cycles were applied until a visible discoloration was achieved.

Artificial saliva was prepared as previously described. 25 Tea stain was prepared by adding one tea bag (Typhoo-One Cup tea bags) per 50 mL of boiling water in a Duran bottle and stirring at room temperature for 5 minutes using magnetic stirrer. After 5 minutes, the tea bags were removed.

### Brushing Experiments

The specimens were fixed in the sample holder and placed in the mechanical brushing device (V8-brushing simulator, JWE GmbH, Germany). Before brushing, the toothbrushes (Signal Kinder Milchzahn toothbrush, for children aged 0–6 years, Unilever, Germany) were wetted with deionized water under standardized conditions for 5 seconds. The toothbrushes were moved in reciprocating motions on the sample surfaces with following parameters: Brushing load 1.5 N and brushing frequency 2.5 Hz. Samples were brushed with horizontal movements for 10, 30, 60, 120, 180, and 300 seconds with toothpaste slurry with the respective dilution degree (1:1, 1:2, 1:4, and 1:8). A continuous movement of the slurry and its presence between toothbrush and specimen was always ensured. The samples were rinsed thoroughly in deionized water after brushing treatment.

### Photographic Documentation and Color Measurements

Photographic images of the enamel samples were taken before the experiment began, after staining and after brushing under standardized conditions using a reflex camera (EOS 600D, Canon Germany GmbH, Germany). Stain removal was calculated from *L* * *a* * *b* * colorimetric measurements (spectrophotometer Konica Minolta Sensing Europe B.V., Germany) conducted on initial, after staining, and after brushing samples using a measurement area of Ø 4 mm. 26 27 28

*
ΔE _1_* : colorimetric difference between brushed and stained specimen
*
ΔE _2_* : colorimetric difference between initial situation and stained specimen

### Statistical Analysis

The results were analyzed by one-way analysis of variance with post hoc Tukey test and Levene's test for analyses of homogeneity of variance (Origin2019b, OriginLab Corporation Company, United States). The level of significance *α* was set at ≤ 0.05.

## Results

**Fig. 2:**
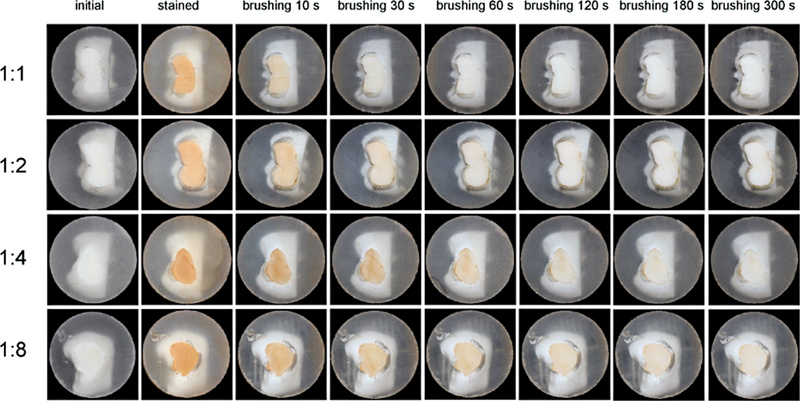
Images of enamel surfaces before staining, after staining, and post-stain removal, brushing was performed for different brushing times (10, 30, 60, 120, 180, 300 seconds) and toothpaste amounts: 1:1 (maximum “full length of brush”), 1:2 (minimum “full length of brush”), 1:4 (“pea-size”), and 1:8 (“grain of rice-size”).

**Fig. 3:**
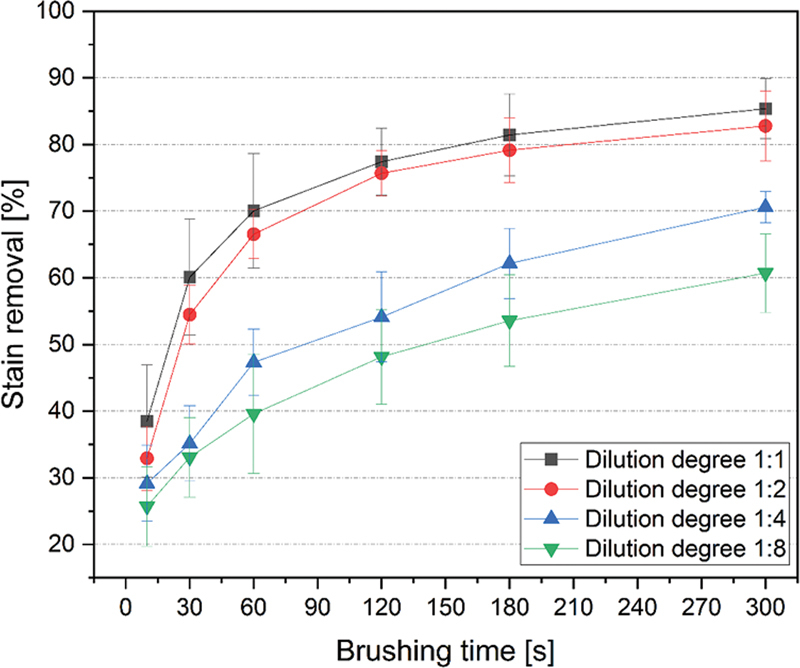
Stain removal efficacy (in % stain removal) for stained enamel specimens after brushing for up to 300 seconds with different toothpaste amounts: 1:1 (maximum “full length of brush”), 1:2 (minimum “full length of brush”), 1:4 ( “pea-size”), and 1:8 (“grain of rice-size”).

In Fig. 2 , exemplary images are shown for one enamel sample per test group and for the respective brushing time. The data of stain removal efficacy determined by the color measurements are presented in Fig. 3 and Table 2 , followed by the results of the statistical analysis ( Table 3 ). For all dilution degrees tested, the stain removal increases with increasing the brushing time. Dilution degree of 1:1 caused at all brushing times the highest stain removal, followed by dilution degree of 1:2, 1:4, and 1:8. While dilution 1:1 removed approximately 40% of stain already after 10 seconds, dilution 1:8 removed approximately 40% of stain after 60 seconds representing a significantly reduced cleaning efficiency of the latter. The maximum cleaning stain removal of the dilution 1:8 was approximately 60% after 300 seconds. The same cleaning efficiency (60%) could be seen in the 1:1 dilution after 30 seconds ( Fig. 3 ).

**Table 2:** Stain removal (mean ± standard deviation, in %) in dependence of brushing time and dilution degree: 1:1 (maximum “full length of brush”), 1:2 (minimum “full length of brush”), 1:4 (“pea-size”), and 1:8 (“grain of rice-size”)

	Dilution degree (toothpaste:water)			
Brushing time	1:1	1:2	1:4	1:8
10 s	38.5 [ ± 8.4]	32.9 [ ± 4.8]	29.1 [ ± 5.7]	25.7 [ ± 6.0]
30 s	60.1 [ ± 8.7]	54.5 [ ± 4.4]	35.1 [ ± 5.7]	33.1 [ ± 6.0]
60 s	70.1 [ ± 8.6]	66.5 [ ± 3.7]	47.3 [ ± 5.0]	39.6 [ ± 9.0]
120 s	77.4 [ ± 5.0]	75.7 [ ± 3.4]	54.1 [ ± 6.7]	48.2 [ ± 7.1]
180 s	81.4 [ ± 6.2]	79.2 [ ± 4.9]	62.1 [ ± 5.3]	53.6 [ ± 6.9]
300 s	85.4 [ ± 4.6]	82.8 [ ± 5.2]	70.6 [ ± 2.3]	60.7 [ ± 5.9]

**Table 3:** *p* -Values are given for the comparison of the stain removal after brushing in dependence of dilution degree and brushing time

	*p* -Values at different brushing times					
Dilution degrees	10 s	30 s	60 s	120 s	180 s	300 s
1:2 vs. 1:1	0.31	0.31	0.75	0.93	0.86	0.69
1:4 vs. 1:1	0.03 a	< 0.0001 a	< 0.0001 a	< 0.0001 a	< 0.0001 a	< 0.0001 a
1:4 vs. 1:2	0.64	< 0.0001 a	< 0.0001 a	< 0.0001 a	< 0.0001 a	< 0.0001 a
1:8 vs. 1:1	0.002 a	0 a	0*	< 0.0001 a	< 0.0001 a	< 0.0001 a
1:8 vs. 1:2	0.13	< 0.0001 a	< 0.0001 a	< 0.0001 a	0 a	< 0.0001 a
1:8 vs. 1:4	0.70	0.91	0.14	0.19	0.03 a	0.001 a

Note: 1:1 (maximum “full length of brush”), 1:2 (minimum “full length of brush”), 1:4 (“pea-size”), and 1:8 (“grain of rice-size”).

a Significant differences.

At brushing with the dilution degree of 1:1 and 1:2, the progression of the stain removal in dependence of the brushing time shows an asymptotic behavior. While the stain removal increases strongly at the beginning (until 120 seconds), the values increase only slightly after a brushing time of 120 seconds ( Fig. 3 ).

At all brushing times, lower (but not significant) stain removal was determined for dilution degree of 1:2 compared to 1:1. At dilution degree 1:1 for all brushing times significantly higher cleaning efficacy was measured compared to dilution degree 1:4 and 1:8. No statistical significance has been determined for the dilution degree of 1:4 and 1:8 after 10, 30, 60, and 120 seconds ( Table 3 ).

## Discussion

The present *in vitro* study shows that the cleaning efficacy of a typical fluoride toothpaste is significantly depended on its amount and on the brushing time. There is a positive correlation between cleaning efficacy and toothpaste amount as well as between cleaning efficacy and brushing time.

Some studies analyzed the effect of different toothpaste amounts (i.e., different toothpaste dilutions) on abrasion. For example, Franzò et al tested both the *in vitro* enamel and dentin wear of two different fluoride toothpastes (with 1450 ppm F ^-^ ) with RDA = 90 (“Toothpaste A”) and RDA = 200 (“Toothpaste B”), respectively. 23 Different toothpaste concentrations were tested, ranging from 0 to 80% (i.e., 0, 14.4, 20, 33.3, 50, 65, and 80%). The enamel wear increased slightly (but not significantly) with increasing toothpaste concentration. Dentin wear, on the other hand, increased significantly with increasing toothpaste concentration. 23 Turssi et al studied the correlation between toothpaste concentration and dentin wear *in vitro* . They found that the dentin wear caused by a toothpaste with a dilution fraction of 1:1 (toothpaste:diluent) is higher than the dentin wear caused by a toothpaste with a dilution fraction of 1:4 (toothpaste:diluent). The diluent they used was deionized water as well as a carboxymethylcellulose-based artificial saliva. 29 Additionally, Wang et al analyzed different commercially available toothpastes in an *in vitro* study and showed that the tested whitening toothpastes had a higher stain removal efficacy than the tested nonwhitening toothpastes (mean values: 59% vs. 27%). 28

To date, however, no study has focused on the stain removal efficacy of varying toothpaste amounts on stained enamel. The strength of the present study is that only the toothpaste amount was varied; all other parameters, for example, the toothbrush type (including filaments) and toothpaste formulation as well as the dilution medium were kept constant. Furthermore, this cleaning efficacy study was carried out using a modified staining model from Lath et al. 24 Please note that a toothbrush for children (0–6 years) was used in this study because of the special recommendations on the amounts of fluoride toothpaste for this age group (see Table 1 ).

The present study clearly demonstrates the influence of different amounts of a test toothpaste with a medium abrasion (RDA: 60.19 ± 1.35) on cleaning efficacy. This study shows that using higher toothpaste amounts and increased brushing time will lead to better cleaning efficacy. However, families have been advised to use smaller amounts of toothpaste for children. For example, the amount of fluoride toothpaste for children is limited to “pea” and “grain of rice” sizes. 20 30 Using a small amount of toothpaste is, however, not only limited to children's oral care. Adults may limit the amounts of toothpaste loaded onto toothbrushes. For example, some people prefer small toothbrush heads. Many electric toothbrush heads are smaller than manual heads and some adults simply prefer to brush with smaller amounts of toothpaste. Due to regulatory reasons it is not possible to simply increase the concentration of all active ingredients to compensate for smaller toothpaste amounts because some ingredients are limited in application concentration due to regulatory reasons. On the other hand, there are other active ingredients, such as calcium phosphate compounds (e.g. amorphous calcium phosphate, β-tricalcium phosphate, hydroxyapatite) 31 which are not limited through regulation in their application concentrations and may even be more beneficial when toothpaste needs to be used in higher amounts. 31 32 33 34 35 36 37 38 39 40

Another argument for using higher amounts (≥ 0.5 g) of toothpaste is that the toothpaste is quickly diluted by saliva during tooth brushing in the oral cavity, 23 and that the brushing time to receive a good cleaning efficacy is reduced compared to smaller toothpaste amounts ( Fig. 3 ).

One could argue that toothpaste is not needed to remove dental plaque. However, this seems to be only true for young and soft biofilms (i.e., plaque accumulation period: 24–96 hours), as shown in a systematic review by Valkenburg et al who analyzed the additional instant effect of toothpastes on mechanical plaque removal. 41 However, clinical results on plaque (and stain) removal from long-term studies are still missing.

The *in vitro* results of this study show that there is a clear influence of toothpaste amount on its cleaning efficacy of clinically relevant plaque and stain. Tellefsen et al, for example, showed in an *in vitro* study that the influence of the toothbrush plus toothpaste on abrasion is more pronounced than the influence of toothbrush alone. 42 Since the abrasion is correlated with the cleaning efficacy, 9 this shows that the toothpaste has a dominant role in cleaning efficacy. In another *in vitro* study, Wang et al showed that brushing with tap water alone (used as a control) is by far less efficient in stain removal than brushing with a toothbrush and toothpaste. 28

It is also important to note that, when using a reduced amount of toothpaste, the absolute amounts of all ingredients, which are present in the oral cavity during tooth brushing, especially active, or medicinal ingredients, are also reduced. Paiva et al, for example, found in an *in situ* demineralization study that the toothpaste amount multiplied by concentration (“intensity of treatment”) is more important than just focusing on the fluoride concentration. 43 This is important as, apart from dental plaque and stain removal, toothpastes are also carriers of remineralization/anticaries agents (e.g. fluorides, 18 hydroxyapatite, 32 33 amorphous calcium phosphates 31 ) and antibacterial agents (e.g. for chemical plaque control and prevention of halitosis). 44 45 Additionally, foaming agents (surfactants) have an important role in cleaning/whitening efficacy as they help to remove hydrophobic compounds from tooth surfaces. 17

This study shows that, in addition to the toothpaste amount, brushing time is also crucial. Cleaning efficacy can be significantly increased by the amount of time taken during tooth brushing. Consequently, patients should be trained to brush their teeth as recommended by many dentists and dental hygienists, and also other dental professionals for at least 2 minutes. Slot et al, for example, show in their systematic review that a longer brushing time using manual toothbrushes increases the mean plaque reduction (i.e., 1 minute tooth brushing: 27 ± 17% plaque reduction; 2 minutes tooth brushing: 41 ± 13% plaque reduction). 46

A limitation of the study is that not all relevant parameters for cleaning efficacy of toothpastes were tested in this study. For example, there are many different toothpaste formulations on the market (e.g., with different types of abrasives and its proportions) and there are different individual brushing habits (e.g., brushing time, brushing load). The influence of these parameters could be tested in future *in vitro* , *in situ* , and *in vivo* studies.

## Conclusion

In this *in vitro* study, the cleaning efficacy of a medium-abrasive, hydrated silica-based toothpaste was analyzed. Note that 1.00 g toothpaste showed for all brushing times a significantly higher cleaning efficacy than 0.25 g toothpaste and 0.125 g toothpaste. It is also important to analyze the cleaning efficacy of different toothpaste amounts in future clinical studies.
